# An Eye to a Kill: Using Predatory Bacteria to Control Gram-Negative Pathogens Associated with Ocular Infections

**DOI:** 10.1371/journal.pone.0066723

**Published:** 2013-06-18

**Authors:** Robert M. Q. Shanks, Viral R. Davra, Eric G. Romanowski, Kimberly M. Brothers, Nicholas A. Stella, Dipti Godboley, Daniel E. Kadouri

**Affiliations:** 1 Department of Ophthalmology, Campbell Laboratory of Ophthalmic Microbiology, University of Pittsburgh, Pittsburgh, United States of America; 2 Department of Oral Biology, University of Medicine and Dentistry of New Jersey, Newark, New Jersey, United States of America; University of Osnabrueck, Germany

## Abstract

Ocular infections are a leading cause of vision loss. It has been previously suggested that predatory prokaryotes might be used as live antibiotics to control infections. In this study, *Pseudomonas aeruginosa* and *Serratia marcescens* ocular isolates were exposed to the predatory bacteria *Micavibrio aeruginosavorus* and *Bdellovibrio bacteriovorus.* All tested *S. marcescens* isolates were susceptible to predation by *B. bacteriovorus* strains 109J and HD100. Seven of the 10 *P. aeruginosa* isolates were susceptible to predation by *B. bacteriovorus* 109J with 80% being attacked by *M. aeruginosavorus.* All of the 19 tested isolates were found to be sensitive to at least one predator. To further investigate the effect of the predators on eukaryotic cells, human corneal-limbal epithelial (HCLE) cells were exposed to high concentrations of the predators. Cytotoxicity assays demonstrated that predatory bacteria do not damage ocular surface cells *in vitro* whereas the *P. aeruginosa* used as a positive control was highly toxic. Furthermore, no increase in the production of the proinflammatory cytokines IL-8 and TNF-alpha was measured in HCLE cells after exposure to the predators. Finally, injection of high concentration of predatory bacteria into the hemocoel of *Galleria mellonella*, an established model system used to study microbial pathogenesis, did not result in any measurable negative effect to the host. Our results suggest that predatory bacteria could be considered in the near future as a safe topical bio-control agent to treat ocular infections.

## Introduction

In an era of increasing antibiotic resistance among bacterial pathogens, the search for new antibiotics and novel treatments for infections caused by these organisms is a priority among researchers. One novel treatment is biological therapy using specific bacteriophage for controlling the infecting pathogen [1]–[3]. Another novel treatment that might hold the potential to treat antibiotic resistant infections are predatory bacteria [4]. Recently, several studies have highlighted the ability of predatory bacteria *Bdellovibrio* spp. and *Micavibrio* spp. to prey on Gram-negative pathogens. Among the pathogens which were evaluated were bacteria associated with oral infections [5]–[7], gastrointestinal infections [8], zoonotic infection [9], pathogens associated with food processing and spoilage [10]–[12], as well as bacteria linked to systemic infections, burns and wounds [13]. Although the data published so far supports the claim that predatory bacteria could be used to control human pathogens, there is still concern regarding the toxic effects of administering large numbers of Gram-negative bacteria as live antibiotics. Therefore, treatment of local infections where the pathogens are easily accessible to topical or locally injected treatment would be ideal candidates to demonstrate a “proof of concept” that infections can be successfully treated with predatory bacteria.

One such local bacterial infection that is treated by direct administration of antibiotic to the site of infection is keratitis, infection of the cornea. Bacterial keratitis can be caused by both Gram-positive and Gram-negative pathogens. Common Gram-negative pathogens associated with keratitis are *Pseudomonas aeruginosa* and *Serratia marcescens* [14]–[16]. Bacterial keratitis is usually localized to an area of the cornea and is treated with antibiotic solutions delivered topically to the eye.

The first step in demonstrating that predatory bacteria can successfully treat bacterial keratitis caused by Gram-negative bacteria is showing that the predatory bacteria can kill Gram-negative bacteria isolated from keratitis cases. Secondly, we must show that the predatory bacteria are non-toxic and non-inflammatory to human cells. In this study we tested whether predatory bacterial species *Bdellovibrio bacteriovorus* and *Micavibrio aeruginosavorus* were able to kill keratitis isolates of *P. aeruginosa* and *S. marcescens,* including antibiotic-resistant isolates, *in vitro*. We also tested whether *B. bacteriovorus* and *M. aeruginosavorus* were cytotoxic and inflammatory to human corneal-limbal epithelial cells (HCLE) *in vitro*. To further test whether these predatory bacteria were pathogenic, we used the *Galleria monella* pathogenesis model to determine whether *B. bacteriovorus* and *M. aeruginosavorus* reduced the viability of the *G. monella* larvae.

## Materials and Methods

### Bacterial strains, and growth conditions

The predatory bacteria used in the study were *Bdellovibrio bacteriovorus* strains HD100, 109J (ATCC 43826) and *Micavibrio aeruginosavorus* strain ARL-13 [17], [18]. Ten *Pseudomonas aeruginosa* and nine *Serratia marcescens* isolates were examined in this study. All clinical isolates were isolated from keratitis patients by Dr. Ritterband at the New York Eye Infirmary and Regis Kowalski at the UPMC Eye Center. Many of these bacteria were fluoroquinolone resistant and previously used in antibiotic efficacy studies [19], [20]. *Pseudomonas aeruginosa* and *Serratia marcescens* were grown with aeration and maintained in LB media. Predator stock-lysates were prepared by co-culturing the predators in the presence of host bacteria suspended in diluted nutrient broth (DNB), a 1∶10 dilution of nutrient broth amended with 3 mM MgCl2 and 2 mM CaCl_2_ [13]. *E. coli* ZK2686 and *P. aeruginosa* UCBPP-PA14 were used as host cells for *B. bacteriovorus* and *M. aeruginosavorus,* respectively. The co-cultures were incubated on a rotary shaker at 30^°^C. Fresh predator cultures were prepared as previously described [13], [21], [22], in brief, 2 ml of overnight-grown host cells (∼1×10^9^ CFU/ml) were added to 2 ml of predatory bacteria taken from a stock-lysate and suspended in 20 ml DNB. The co-cultures were incubated for 24 hrs at 30°C to reach ∼1×10^8^ PFU/ml predator’s cells. At this point, the lysates were filtered through a 0.45 µm Millex-HV pore-size filter (Millipore, Billerica, MA) in order to remove any residual host cells (harvested predator).

### Predation Experiments

Predation experiments were conducted as previously described [13]. Five ml co-cultures were prepared by adding 0.5 ml of washed host cells to 0.5 ml of freshly harvested predator bacteria in DNB media. The cultures were incubated at 30°C for 48 hrs. The capability of *B. bacteriovorus* and *M. aeruginosavorus* to prey was evaluated by the reduction in prey cell viability in the predator co-cultures. Cell viability was measured by dilution plating and CFU enumeration at 24 and 48 hrs. Each co-culture was conducted twice in triplicate.

### Cytotoxicity assays

*B. bacteriovorus* and *M. aeruginosavorus* were prepared as described above using 5 ml of washed host cells and 5 ml of freshly harvested predator in 50 ml DNB media. The co-cultures were incubated for 24 and 36 hrs for *B. bacteriovorus* and *M. aeruginosavorus,* respectively. Thereafter, the lysates were filtered four times through a 0.45-µm Millex-HV pore-size filter to remove any residual host cells. The filtered harvested lysate was washed twice by centrifugation, 15,000 rpm for 30 min, and resuspended in 2 ml of DNB. Aliquots of the predator preparation was removed and plated on agar plates, to confirm that the samples are free from host cells. Samples were also taken to determine predator concentration using standard double-layered agar method [23]. Purifications were conducted on 3 and 4 separate occasions for *M. aeruginosavorus* and *B. bacteriovorus*, respectively.

Cytotoxicity assays were conducted as described [24] with some modifications, Human corneal-limbal epithelial (HCLE) [25] cells were cultured in 24-well plates until they were confluent. HCLE cells were grown in Keratinocyte serum-free medium (KSFM) with L-Glutamine, supplemented with 25 µg/ml BPE, 0.2 ng/ml EGF, and 1 mM CaCl_2_. HCLE cells were seeded without antibiotics to prevent interference of antibiotics in subsequent assays. The plates were incubated in an incubator at 37°C with 5% CO_2_. The wells were washed 3 times using PBS, pH 7.4 (Sigma-Aldrich, St. Louis, MO) and 450 µl of KSFM media was added to each well. Thereafter, wells were inoculated with 50 µl of each predator prep (∼0.2–1.1×10^9^ PFU/well for *B. bacteriovorus* strains and ∼2×10^8^ PFU/ well for *M. aeruginosavorus*) or predator free DNB control for maximum viability. Other controls included 0.25% of triton X-100 to measure total killing and 50 µl of DNB washed *P. aeruginosa* PA14 (∼2.5×10^7^ CFU/well) as a positive control for bacterial cytotoxicity. Cell cultures were incubated for 4 and 24 hrs. After the incubation, aliquots of medium were removed from each well, centrifuged to remove bacteria, and stored at –20°C for subsequent pro-inflammatory cytokine analysis. The cells were then washed three times with PBS. Alamar Blue viability reagent (Invitrogen) in KSFM containing amikacin (10 µg/ml) was added to each well (500 µl/well) to assess cell viability. Fluorescence was measured after 1.5 hrs of incubation using a Synergy 2 microplate reader (Biotek) at 500/27 nm excitation and 620/40 nm emission wavelength. Experiments were conducted four times using *B. bacteriovorus* and three times using *M. aeruginosavorus*. Each experiment was conducted in quadruplicate (4 cell culture wells).

### Cytokine analysis

HCLE supernatants were collected at 4 and 24 hrs post bacterial exposure. Four biological samples were used for ELISA and also tested on two different days with a different harvest sample set. IL-8 and TNFα ELISAs were run on the 4 and 24-hour sample sets according to manufacturer’s instructions (for IL-8, R & D Systems^®^; for TNFα, Thermo Scientific Pierce Biotechnology). Upon completion of the assay, samples were read according to the manufacturer’s instructions on a Synergy 2 plate reader (BioTek). Samples were graphed and statistical analysis was performed using GraphPad Prism 5 using one-way ANOVA with Tukey’s post-hoc test.

### Toxicity assay in *Galleria mellonella* invertebrate infection model

Viability experiments were conducted as described previously [26] with some modifications. *Galleria mellonella* larvae were obtained from New York worms (New York Worms, Glen Cove, NY). Larvae were in their final instar stages and had equal size and weight (330±30 mg). *B. bacteriovorus* strains, 109J, HD100, and *M. aeruginosavorus* ARL-13 were grown and concentrated as descried for the cytotoxicity assays. The predators were suspended in phosphate buffered saline (PBS), pH 7.4 (Sigma-Aldrich, St. Louis, MO). Final predator concentration 2×10^9^ PFU/ml. Five microliters of each sample was injected into the hemocoel of each larva via the last left proleg using a Hamilton 25 µl syringe and 30.5-gauge needle. Prior to use, the syringes were sterilized using bleach. The syringes were cleaned with 70% alcohol and distilled water and the needles were changed between every sample. In addition to the predators, worms were also injected with 5 µl of PBS buffer (negative control) and 5 µl of 8×10^4^ CFU/ml *P. aeruginosa* PA14 (positive control). After injection, the worms were incubated at 30^°^C and the numbers of live larvae were scored for 11 days. Larvae were considered dead when they display no movement in response to gentle shaking of the dish or touching with a pipette tip. Six petri dishes containing 5 worms were assigned to each experimental and control groups (30 worms total for each sample).

### Statistical Analysis

Graphpad Prism 5 software was used to perform statistical analysis. This analysis consisted of One-way ANOVA with Tukey’s multiple comparison test.

## Results

### Predation by B. bacteriovorus and M. aeruginosavorus

When exposed to the predators, all of the isolates were found to be susceptible to at least one predator. All *S. marcescens* isolates were found to be susceptible to predation by both *B. bacteriovorus* 109J and HD100, with cell reduction ranging from 1.7 log_10_ to greater than 5 log_10_, compared to the initial cell concentration and the predator free control (Table 1). 100% of the tested *P. aeruginosa* isolates were reduced by *B. bacteriovorus* HD100. However, only 70% of the isolates were reduced by the 109J strain. Eight of the 10 *P. aeruginosa* isolates were reduced by *M. aeruginosavorus* with a greater than 2-log_10_ reduction measured for 87% of the predation positive strains (Table 1). It was previously shown that *M. aeruginosavorus* ARL-13 is able to use *P. aeruginosa* as prey; however, it is unable to utilize *S. marcescens* [13], [22]. Therefore, in this study, *M. aeruginosavorus* was not tested on *S. marcescens.*

**Table 1:** Predation of *S. marcescens* and *P. aeruginosa* ocular isolates by predatory bacteria.

Bacteria and strain	Time_0_ (CFU/ml)	Control (Log_10_ change)	*B. bacteriovorus* 109J(Log_10_ change)	*B. bacteriovorus* HD100 (Log_10_ change)	*M. aeruginosavorus* ARL-13(Log_10_ change)
***Serratia marcescens***					
K912	1.25×10^8^	+0.74±0.46	–1.7±0.15	–2.62±0.03	na
K1064	9.43×10^8^	–0.04±0.06	–2.63±0.06	–4.55±0.10	na
K1097	4.32×10^8^	+0.09±0.13	–3.56±0.07	–4.17±0.10	na
K1154	5.64×10^8^	–0.09± 0.05	–3.91±0.06	–4.24± 0.01	na
K1885	3.48×10^8^	+0.22±0.02	–3.7±0.24	–4.6±0.19	na
K1496	6.06×10^8^	+0.07±0.10	–3.74±0.01	–5.28±0.08	na
K2093	3.91×10^8^	+0.07±0.08	–2.88±0.06	–3.94±0.2	na
K2119	1.25×10^8^	+0.24±0.11	–3.5±0.24	–5.48±0.06	na
K2282	1.28×10^8^	+0.89±0.03	–4.39±1.13	–3.05±0.29	na
***Pseudomonas aeruginosa***					
PaA	3.56×10^8^	+0.29±0.18	–4.97±0.13	–3.5±0.19	–1.10±0.50*
PaB	5.00×10^8^	+0.23±0.03	–3.67±0.01	–2.74±0.22*	+0.12±0.08^Ψ^
PaC	7.03×10^8^	+0.07±0.06	–2.13±0.15	–3.91±0.03	–2.98±0.08
PaD	3.26×10^8^	–0.69±0.02	–2.06±0.27	–3.66±0.16	–2.86±0.21*
Pa16	8.28×108	–0.07±0.03	–3.58 ±0.06	–2.18±0.24*	–0.19±0.09
K2418	4.91×10^8^	+0.25±0.10	+0.18±0.06	–3.01±0.42*	–2.74±0.40
K2409	1.07×10^9^	–0.08±0.03	–4.18±0.14	–4.48±0.06	–2.03±0.16*
K2222	8.51×10^8^	–0.01±0.26	–2.78±0.11	–2.19±0.43	–2.85±0.04*
K2414	7.26×10^8^	+0.16±0.08	–0.04±0.33^Ψ^	–1.16±0.23	–2.85±0.10*
K2421	8.38×10^8^	+0.29±0.10	–0.29±0.21	–2.61±0.22	–3.51±0.43

Co-cultures were prepared by adding host cells to harvested predator cells (∼1×10^7^ PFU final concentration) or predator free control. Values represent the maximum Log_10_ change measured following 24 or 48 (*) hrs of incubation (compared to t_0_). Each experiments was conducted twice in triplicate yielding similar results. Value representing the mean and standard error from one representative experiment.

**n.a.**- not applicable.

**Time 0**- initial host concentration (CFU/ml).

**+**  =  Increase in host numbers.

–  =  Decrease in host numbers.

### Cytotoxicity B. bacteriovorus and M. aeruginosavorus to HCLE cells

**Figure 1:**
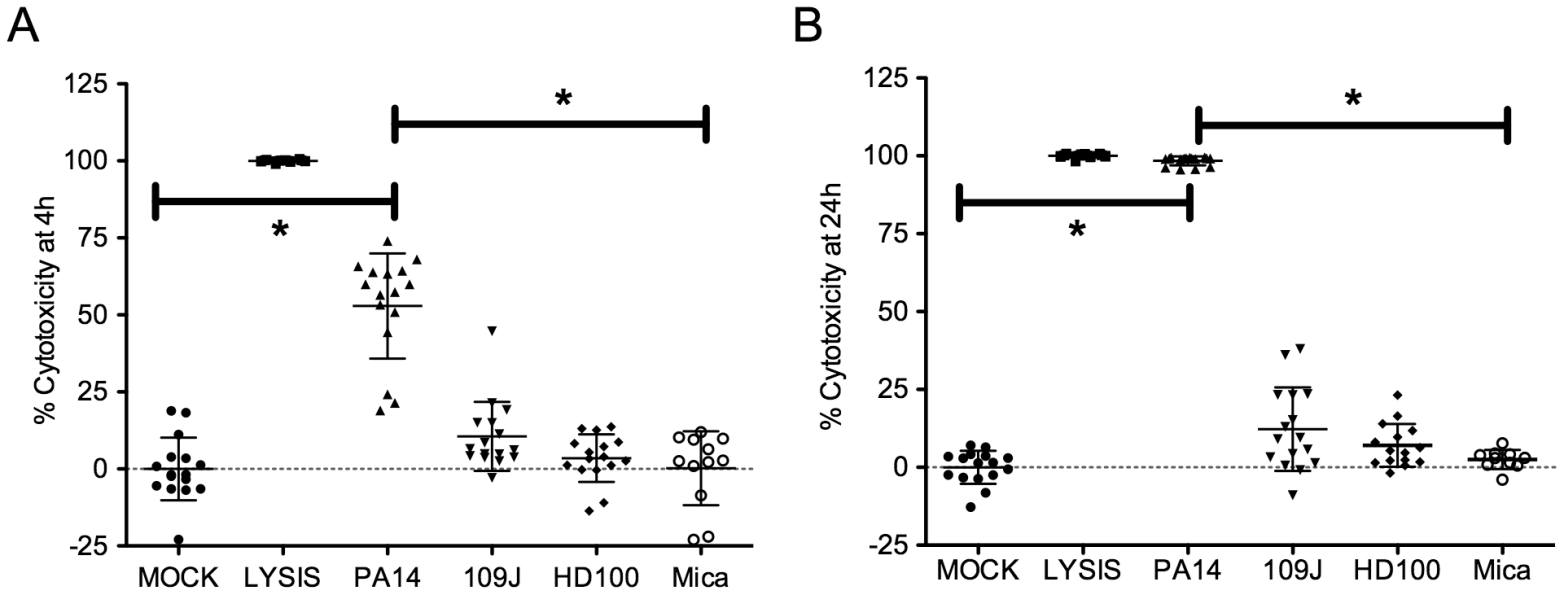
Cytotoxicity to human corneal-limbal epithelial cells *in vitro*. Alamar blue vital stain was used to measure cytotoxicity from positive control *P. aeruginosa* strain PA14 (average MOI  =  111), detergent lysis (LYSIS), medium only negative control (MOCK), and experimental strains *B*. *bacteriovorus* strain 109J (average MOI  =  4720), *B. bacteriovorus* strain HD100 (average MOI  =  1039), and *M. aeruginosavorus* (Mica, average MOI  =  853). HCLE viability was measured after 4 h (A) and 24 h (B) of exposure. Total independent data points from 4 experiments are shown. Asterisks indicate significant differences (p<0.001, ANOVA with Tukey's post-test). Only PA14 was significantly different than MOCK. Error bars indicate one standard deviation.

As a first step towards judging the suitability of predatory bacteria for ocular infections, we tested whether *B. bacteriovorus* and *M. aeruginosavorus* were cytotoxic to HCLE cells *in vitro*. Bacteria were co-incubated with HCLE cells at an MOI of ∼100 with a known cytotoxic *P. aeruginosa* strain [27] as a positive control, and >800 for each of the predatory bacteria. Bacteria and HCLEs were co-incubated for 4 and 24 hours, then bacteria were removed and HCLE cells were tested for viability using the fluorescent vital stain alamar blue. Whereas *P. aeruginosa* was highly cytotoxic at both time points, the predatory bacteria were not significantly different from the mock at either 4 or 24 hrs (p>0.05, ANOVA, with Tukey’s post-test) (Figure 1).

### Production of pro-inflammatory cytokines following exposure to predatory bacteria

**Figure 2:**
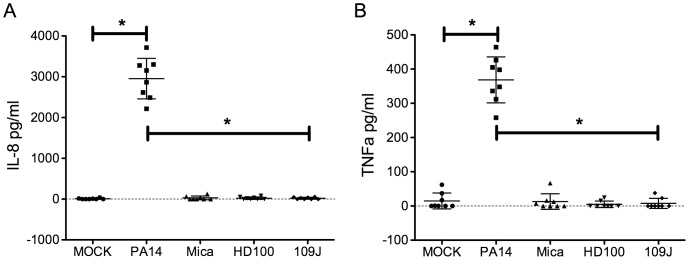
Inflammatory response of human corneal-limbal epithelial cells to predatory bacteria *in vitro*. Pro-inflammatory cytokines IL-8 (panel A) and TNF-α (panel B) were measured using ELISA assays. Cell supernatants taken from HCLE cells after 4 hrs of incubation with positive control *Pseudomonas aeruginosa* strain PA14 (average MOI  =  111), detergent lysis (LYSIS), medium only negative control (DNB), and experimental strains *B. bacteriovorus* strain 109J (avgerage MOI  =  4720), *B. bacteriovorus* strain HD100 (average MOI  =  1039), and *M. aeruginosavorus* (Mica, average MOI  =  853). Total independent data points from 2 experiments are shown. Asterisks indicate significant differences (p<0.001, ANOVA with Tukey's post-test). Only PA14 was significantly different than MOCK. Error bars indicate one standard deviation.

Because the predatory bacteria used in this study are Gram-negative bacteria, we predicted that they may cause adverse inflammatory effects upon exposure to ocular cells. Supernatants of HCLE cells co-incubated for four hrs with *B. bacteriovorus* and *M. aeruginosavorus* in the above noted cytotoxicity studies and were analyzed for proinflammatory cytokines IL-8 and TNF-a. These cytokines were chosen because they are expressed by ocular surface cells exposed to bacteria [28], [29]. Whereas the positive control, *P. aeruginosa*, elicited a strong and significant induction of both cytokines, neither IL-8 nor TNF-a was found to be elevated above the mock negative control in HCLE supernatants co-incubated with any of the three predatory bacteria (Figure 2A–B). The same pattern was observed after co-incubation of the predatory and positive control bacteria at 24 hrs for IL-8; however, TNF-a levels were undetectable (data not shown).

### *In vivo* effect of predatory bacteria on *G. mellonella*

*G. mellonella* was recently recognized as a suitable host model system to study microbial pathogenesis and innate immunity [30]–[34]. The *G. mellonella* system generally demonstrates a positive correlation between virulence factors found in mammals to those isolated in the insect, emphasizing the ability to utilize the system to bridge between in vitro studies and vertebrates [26]. In order to further evaluate the potential risk of using predatory bacteria *G. mellonella* worms were exposed to high concentrations of each predator. All of the worms injected with *P. aeruginosa* PA14 (4×10^2^ CFU/worm) were nonviable 24 hrs post-injection. However, worms injected with PBS, *B. bacteriovorus* 109J, HD100 (1.1×10^7^ PFU/worm) and *M. aeruginosavorus* ARL-13 (0.9×10^7^ PFU/worm) were all viable 24 hrs post-injection. 11 days post-injection, the viability of the worms were 96.6%, 100%, 96.6% and 93.3% viability for the control, *B. bacteriovorus* 109J, HD100 and *M. aeruginosavorus* ARL-13, respectively. Furthermore, no change in larva pigmentation was observed in the predator-infected worms (Data not shown). In *G. mellonella* the change in color indicates melanization caused by the host immune response to the microbial challenge [26]. Thus, based on our finding it could be concluded, that unlike other pathogens, predatory bacteria do not provoke an aggressive innate immune response when injected.

## Discussion

In this study, we have demonstrated that *M. aeruginosavorus* ARL-13 is able to prey on clinical isolates of *P. aeruginosa* isolated from ocular infections. This finding is in line with earlier reports regarding the host specificity of this predator and its ability to attack *P. aeruginosa* [13], [22], [35]. Our data also suggest that both *B. bacteriovorus* 109J and HD100 are capable of using *S. marcescens* as a host. This finding is in agreement with a study reporting a 3 log_10_ reduction in cell viability of a non clinical isolate of *S. marcescens* following predation by *B. bacteriovorus* 109J [13]. In addition, *B. bacteriovorus* 109J and HD100 were able to prey on *P. aeruginosa.* However, the ability of the HD100 strain to attack was broader than that of 109J, preying on 100% and 70% of the isolates, respectively. The narrower ability of *B. bacteriovorus* 109J to prey on *P. aeruginosa* is aligned with a recent report in which *B. bacteriovorus* 109J was able to reduce only 1 out of 4 *P. aeruginosa* examined strains [5], as wall as earlier findings showing a limited ability of some *B. bacteriovorus* to prey on *P. aeruginosa* [8], [36]. The different host- and intra-species strain- specificity demonstrated by *Bdellovibrio* spp. and *Micavibrio* spp. is well documented. Furthermore, predation ability was found to be specific to the *B. bacteriovorus* strain used, with different *B. bacteriovorus* strains demonstrating unique host specificity [8], [13], [22], [35]–[38]. As the mechanisms that define the predator’s host specificity are not fully known, we could only speculate on the reason why certain bacteria are recognized as a host while others are not.

A major concern that needs to be addressed when evaluating the potential use of predatory bacteria as topical live-antibiotics are the risks associated with applying Gram-negative microorganisms to human cells. To this end, we have conducted cytotoxicity assays in which HCLE cells were exposed to high concentrations of the predators. Our data indicate that predatory bacteria are significantly less cytotoxic than the control *P. aeruginosa*. Low cytotoxicity was observed even after an extended exposure period. Although only a small number of studies regarding the safety of using predatory bacteria were conducted, the current data does support the claims that predatory bacteria could be considered safe. In a review article published by Dwider at al [4] the authors cited a study in which *Bdellovibrio* was injected into mice rabbits and guinea pigs and were found to be non pathogenic to the animals [39]. In a separate study conducted by Lenz and Hespell [40], the investigators attempted to grow the predatory bacteria *B. bacteriovorus* 109J, *Bacteriovorax stolpii* UKi2 and *Peredibacter starrii* A3.12 in the presence of eukaryotic cells. It was concluded that predatory bacteria are unable to grow on hamster kidney cells, mouse liver cells and bovine mammary gland cells. Furthermore, the predators did not grow within rabbit ova, following injection, nor were they able to grow in media containing rabbit ova extracts. Thus, it seems that mammalian cells could not be used by the predators as prey and could not support predator proliferation in the absent of a Gram-negative host. The inability of the predator to grow and establish itself in the intestinal microflora was shown in a study in which *Bdellovibrio* strain MS7 was fed to Channel catfish, northern leopard frogs, and mice. The predator viability also declined when inoculated into rabbit ileal loops. As *Bdellovibrio* could not proliferate *in vivo* it reduces the risk of permanent establishment within the mammalian host, rendering the predator, in the study, as nonpathogenic [41].

In a recent study conducted at the University of Nottingham [9], the *in vivo* effect of *Bdellovibrio* in a poultry vertebrate model was examined. It was found that *B. bacteriovorus* HD100, which was orally administered to chicks, caused no negative health effects on the birds. Furthermore, the authors were not able to recover viable *Bdellovibrio* from the gut flora, fecal matter or drinking water of the predator-inoculated birds, concluding that the risk of spreading predatory bacteria during treatment is low. *Bdellovibrio* treatment was also found to improve the well being of the birds colonized with *Salmonella* Enteritidis in the therapeutic trail.

An additional concern of applying live predatory bacteria is the risk of inadvertently causing inflammation which in could inhibit wound healing and increase the risk of tissue damage. In this study we have demonstrated that exposure to high doses of the predators did not elevate the production of the proinflammatory cytokines IL-8 and TNF-alpha by HCLE cells. Experimental evidence supports that neutrophils attracted by the bacteria-induced inflammation are a major cause of scarring and tissue damage associated with vision loss in keratitis [42]. The low cytotoxic activity of *B. bacteriovorus* HD100 LPS and its reduced ability to induce TNF-alpha and IL-6, compared to an *E. coli* control, was previously reported in a study using a human macrophage cell line [43]. The authors attributed their findings to the unique structure of the *B. bacteriovorus* LPS Lipid A molecule. Unlike Lipid A from many Gram-negative bacteria that contain negatively charged phosphate groups, the *B. bacteriovorus* Lipid A molecule has α-D-mannose residues which reduced its affinity to LPS receptors thereby lowering inflammation. Our data confirm that *B. bacteriovorus* HD100 and 109J do not enhance proinflammatory cytokines production. Our data also demonstrate, for the first time, that as reported for *B. bacteriovorus o*, *M. aeruginosavorus* also does not enhance inflammation.

Although, our data show that predatory bacteria have little or no adverse effect when applied to human cell cultures, we were interested to evaluate the effect of predatory bacteria *in vivo*. To address whether predatory bacteria a tolerated by eukaryotic cells *in vivo*, a *G. mellonella* microbial pathogenesis model was selected. Although, we did not measure the viability of the predators within the worm over time, we might still conclude that injecting relatively high doses of predatory bacteria do not provoke any measurable toxic or harmful effect to the worm. *G. mellonella* is recognized as a suitable host model system to study the pathogenesis of both bacteria and yeast and was used to examine pathogenic attributes of many human pathogens including pathogens associated with eye infections [26], [30], [32]. These studies established the use of *G. mellonella* for a variety of applications such as: examining microbial pathogenicity and lethality, evaluating microbial growth and proliferation, isolating virulence factors, and inspecting putative virulence mechanisms [30], [32]-[34], [44]. Since the innate immune systems of mammals and insects have several features in common [31], *G. mellonella* could also be used as a model system for studying the host innate immune response to microbial infection as well as identifying microbial virulence factors that mediate the immune response [33], [45].

The potential use of predatory bacteria as a bio-control agent to treat eye infections was first suggested some 40 years ago. In a study conducted in 1972 [46] the “pro-biotic” ability of *E. coli* and *B. bacteriovorus* to impact the pathogenesis of *Shigella flexneri* in animal modules was examined. It was shown that the *B. bacteriovorus* was able to reduce the severity of keratoconjunctivitis induced by *S. flexneri* in a rabbit keratoconjunctivitis model. The simultaneous inoculation of *Bdellovibrio* with *S. flexneri* was able to prevent the development of the infection. The rate of development of typical keratoconjunctivitis was also decreased when *Bdellovibrio* was administered within 48 hrs of the initial *S. flexneri* infection. In a more recent study, the ability of *B. bacteriovorus* 109J to inhibit growth of and reduce the adherence of *Moraxella bovis* to Madin-Darby bovine kidney (MDBK) cells, used to mimic bovine keratoconjunctivitis, was confirmed [47]. The ability of *Bdellovibrio* to survive and prey in human fluids was also demonstrated in an experiment in which *B. bacteriovorus* 109J was abele to significantly reduce biofilms of *Aggregatibacter actinomycetemcomitans* in the presence of human saliva that contains many of the same antimicrobial compounds as do tears [5].

In conclusion, our work demonstrates that predatory bacteria have the ability to attack “real-life” Gram-negative human pathogens associated with ocular infection. Furthermore, *in vitro* studies had revealed that the presence of high concentrations of predatory bacteria don’t appear to be harmful to human cells. These findings, coupled with the ability of predator bacteria to prey in conditions that might be encountered in the eye, emphasize the potential use of applying predator bacteria as a topical agent to treat eye infections caused by pathogens which are resistant to traditional antimicrobials. The efficacy of predatory bacteria to control infection using *in vivo* models of ocular infection should be the focus of future studies. The long-term goal is to develop a topical predator bacteria product, which might include a single or multispecies predator bacteria mix and could be used alone or in concert with traditional antimicrobial therapies.
